# Participants’ perceptions and understanding of a malaria clinical trial in Bangladesh

**DOI:** 10.1186/1475-2875-13-217

**Published:** 2014-06-04

**Authors:** Debashish Das, Phaik Yeong Cheah, Fateha Akter, Dulal Paul, Akhterul Islam, Abdullah A Sayeed, Rasheda Samad, Ridwanur Rahman, Amir Hossain, Arjen Dondorp, Nicholas P Day, Nicholas J White, Mahtabuddin Hasan, Aniruddha Ghose, Elizabeth A Ashley, Abul Faiz

**Affiliations:** 1 Mahidol Oxford Tropical Medicine Research Unit, Faculty of Tropical Medicine, Mahidol University, 420/6 Rajvithi Rd., Bangkok 10400, Thailand; 2 Centre for Tropical Medicine, Nuffield Department of Clinical Medicine, University of Oxford, Oxford, UK; 3 Chittagong Medical College Hospital, Chittagong, Bangladesh; 4 Cox’s Bazar Medical College, Cox’s Bazar, Chittagong, Bangladesh; 5 Ramu Upazila Health Complex, Cox’s Bazar, Chittagong, Bangladesh; 6 Shaheed Suhrawardy Medical College, Dhaka, Bangladesh; 7 Dev Care Foundation, Chittagong, Bangladesh

**Keywords:** Consent, Ethics, Clinical trials, Bangladesh, Malaria

## Abstract

**Background:**

Existing evidence suggests that there is often limited understanding among participants in clinical trials about the informed consent process, resulting in their providing consent without really understanding the purpose of the study, specific procedures, and their rights. The objective of the study was to determine the subjects’ understanding of research, perceptions of voluntariness and motivations for participation in a malaria clinical trial.

**Methods:**

In this study semi-structured interviews of adult clinical trial participants with uncomplicated falciparum malaria were conducted in Ramu Upazila Health Complex, in Bangladesh.

**Results:**

Of 16 participants, the vast majority (81%) were illiterate. All subjects had a ‘therapeutic misconception’ i.e. the trial was perceived to be conducted primarily for the benefit of individual patients when in fact the main objective was to provide information to inform public health policy. From the patients’ perspective, getting well from their illness was their major concern. Poor actual understanding of trial specific procedures was reported despite participants’ satisfaction with treatment and nursing care.

**Conclusion:**

There is frequently a degree of overlap between research and provision of clinical care in malaria research studies. Patients may be motivated to participate to research without a good understanding of the principal objectives of the study despite a lengthy consent process. The findings suggest that use of a standard consent form following the current ICH-GCP guidelines does not result in achieving fully informed consent and the process should be revised, simplified and adapted to individual trial settings.

## Background

Regulatory authorities and ethical guidelines emphasize the need for research subjects to understand the informed consent process [1-4]. Ensuring that appropriate information is disclosed to potential research participants in an understandable manner in order to empower them to make a voluntary decision is one of the key ethical requirements in biomedical research [4].

The ICH guideline on Good Clinical Practice lists 20 items that should be explained in the participant information sheet and consent form [5]. Achieving genuine informed consent is becoming more difficult as patient information sheets get longer and clinical trials become increasingly complicated with the continued expansion of research into new areas such as molecular biology and genetics. The vocabulary to describe all these new techniques does not exist in all languages. Despite this, ethics committees put a lot of emphasis on obtaining informed consent for genetic testing or the storage of samples for future research.

It has been recognized widely by ethicists and medical researchers that trial participants may not completely understand the information they are being provided with, and may have very little idea of what they are actually consenting to when they agree to be involved in biomedical research. This shortfall in comprehension is widespread and not only a problem restricted to less developed countries [6-10]. However, the decision to participate in research in resource constrained settings is more likely to be swayed by other factors since, in general, potential participants are more likely to come from vulnerable groups in the community [6]. For example, they are more likely to have lower educational status, more linguistic barriers, lower socio-economic conditions, and limited experience of health care and research which will impact on their understanding and perception of research and eventually the entire informed consent process. Understanding of consent remains a relatively under-researched area in developing country contexts.

### Context and rationale

A study site was established in Ramu Upazila Health Complex (UHC), Cox’s Bazar, Bangladesh, as part of a multicentre clinical trial by the “Tracking resistance to Artemisinin Collaboration – TRAC” (Clinical trial registration number NCT01350856) in patients with uncomplicated falciparum malaria. The sub-district hospital provides primary health care services for 167,480 inhabitants in Ramu, who are predominantly illiterate (average literacy rate 34%) and usually make a living by farming, tree plantation and physical labour (1991 Bangladesh census). In 2011, malaria accounted for 6% of inpatient hospital admissions in Ramu UHC (Ministry of Health and Family Welfare Health Bulletin 2012). Malaria, one of the common causes of febrile illness, is often manifested by headache, malaise, muscle-ache, nausea, vomiting, anorexia, fever with chills and rigors, or in its severe form by coma and convulsions. The TRAC study involved an inpatient hospital stay, which would not have been otherwise indicated for the routine medical care for the treatment of uncomplicated malaria. Patients were hospitalized in order to monitor the decrease in parasitaemia precisely as a marker of artemisinin susceptibility which was the main end-point of the study. The study also involved frequent blood sampling from subjects and storage of samples for future research such as measurement of drug concentrations, and in vitro, molecular and parasite genetic assays. The complex nature of the trial in the context of a resource-poor setting prompted the researchers to design a pilot sub-study on the informed consent process.

### Objectives

The objective of the current study was to assess the participants’ understanding of research, their perception of voluntariness and to determine factors influencing their decision making process in regards to participation in a clinical trial in Bangladesh. The study also aimed to get a better understanding of participants’ concerns related to specific procedures (e.g. frequent blood sampling, long hospital admission) and future use of samples, as well as identifying the barriers to achieving study comprehension in a developing country context.

## Methods

### Patients and study procedures

In this qualitative study, adult (>18 years) patients with malaria that consented to the TRAC clinical trial in Ramu Upazila Health Complex, Cox’s Bazar, Bangladesh were interviewed between July and October 2012. The clinical trial was already underway when approval for this study was obtained and 13 patients out of a total of 56 had been recruited. Potential participants for this sub-study were selected sequentially by purposive sampling from the TRAC study ward commencing with the fourteenth study subject. Two of the authors (FA and DD) approached individuals and briefly invited them to take part in an in-depth interview. If individuals expressed interest, they were accompanied to a private room where they received further information about the study. If they agreed to be interviewed, then verbal consent in the local language (Bangla) was obtained and documented on the audiotape. It was made clear that participation in this sub-study was solely voluntary and refusing consent for this would not jeopardize clinical care and treatment in the main TRAC trial. The interview and entire conversation between the respondent and the interviewer was recorded using a digital tape recorder for the purpose of future translation and transcription. The information collected from interviewees was anonymous and confidentiality was strictly maintained. Ethical approval was obtained from the Oxford Tropical Medicine Ethics Committee, and Bangladesh Medical Research Council.

### Data collection tools

A semi-structured interview guide was designed to capture patients’ perceptions and understanding of research, the informed consent process and TRAC trial specific procedures (see Additional file 1). The questions were grouped in categories according to the study objectives. The interviews were conducted by a researcher during hospital admission (on the second or third day after enrolment) once the participants’ acute symptoms of malaria had resolved. The interviewers had been trained on the purpose of the study, data collection tools and techniques. The interview was recorded using a digital tape recorder if permission was given by the respondent.

### Informed consent process

Three study nurses were responsible for obtaining informed consent to participate in the TRAC clinical trial. Before the trial, in addition to the TRAC protocol procedures, the research team went through a one-day Good Clinical Practice (GCP) training. They also received hands-on training on how to obtain written informed consent from study subjects. This included explanation of the utility and obligations of information provision for potential research participants according to international codes of ethics.

### Statistical analysis

Adult patients enrolled in TRAC trial at Ramu UHC were selected sequentially for in-depth interviews using a semi-structured interview guide. The conversation between a subject and an interviewer was recorded. Literatim transcripts of the interviews were translated into English. All transcripts were reviewed and major themes were highlighted and discussed by the researchers. An arbitrary sample size of 16 was selected for this exploratory study which was considered to provide sufficient data to explore the study questions.

## Results

A total of 16 of the 56 patients enrolled in the clinical trial were interviewed (29%). Three adult male patients from the same villages as other participants recruited and completed follow up in the clinical trial but refused to participate in this qualitative sub-study. No reason was given for refusal. The average duration of the interview was about 30 minutes. The respondents were predominantly male (88%) from Bangali ethnic background (100%) and the median age was 27 years (range, 18–51). This reflected the age and gender distribution of the patient recruitment to the clinical trial, a consequence of the fact that in Asia malaria is mainly an occupational disease of young men. More than three quarters of the interviewees (13/16) were illiterate (i.e. could not read or write or could not read but were able to write or sign their name). All participants lived in villages and three fourths worked either as farmers or day labourers. The average monthly income of this subject cohort was approximately 6500 Bangladeshi Taka (BDT) ranging from 3000–12500 BDT (1 USD = 80 BDT). In regards to languages spoken, the majority of participants (88%) could speak Chittagonian (the local dialect) in addition to Bangla (the official language of Bangladeshi people). The characteristics of the participants are given in Table 1.

**Table 1:** Characteristics of the study participants (N = 16)

**Variable**	**Frequency (%)**
**Gender**	
Male	14 (88)
Female	2 (12)
**Age (years), median (range)**	27 (18–51)
**Ethnicity**	
Bangali	16 (100)
**Education level**	
Cannot read or write	2 (12)
Cannot read but can write name	11 (69)
Primary schooling	3 (19)
**Occupation**	
Farmer	3 (19)
Day labourer	9 (56)
Govt. or private employee	2 (12)
Household work	2 (12)
**Language**	
Bangla only	2 (12)
Both Chittagonian and Bangla	14 (88)
**Monthly income (BDT)**	
< = 5,000	7 (44)
> 5,000	9 (56)

*Abbreviation:* BDT, Bangladeshi Taka.

### Perceptions of research

None of the sub-study subjects had previous experience of participation in clinical research. The vast majority had limited experience with routine medical care provided in hospitals. Half of the patients had never had malaria before. However, their responses demonstrated a reasonably good understanding of malaria. This general malaria awareness might be explained by the activities of the National Malaria Control Programme (NMCP), in collaboration with local partners (such as Mukti, a NGO in Ramu) providing diagnostics, treatment services and health education at village level in malaria endemic areas in Bangladesh.

‘I went to Alikadam, Bandarban hill and stayed there for 24 days to collect wood. I took bed net with me but I was bitten by mosquitoes during day time and also at night as it was rainy season. Nothing happened there, but upon arrival at home, I started feeling sick after 2 days along with fever, chills & rigor, muscle-ache and came to Ramu hospital on the next day. ….The doctor sent me for blood testing. Then I was told that I had malaria’.

In contrast, the subjects showed relatively poor actual understanding of the research activities. The TRAC trial involved specific procedures, i.e., repeated blood sampling and inpatient hospital stays which are not practised in routine hospital management of uncomplicated malaria. None of the subjects interviewed mentioned the purposes of storage of samples for future drug measurement and genetic studies. Regarding blood sampling, the perceptions were mainly:

*‘I talked to my colleague over phone. He asked me about the amount and frequency of blood taken. I said about a spoonful each time. He asked me to continue and assured that this should be fine, no problem.* Prompted by the interviewer *- OK, so your friend reassured you. But why did they take blood samples from you?The respondent mentioned“to test my blood, to see the germs and eventually, to make me well”.*

Furthermore, three out of 16 subjects justified their inpatient hospital admission by having severe malaria which was a wrong perception. Others mentioned the purpose of hospital admission was to ensure cure from the illness. In terms of anti-malarial treatment, the vast majority had a clear idea of the duration of treatment. Almost all respondents thought they understood the reason they were asked to participate in the research. However, none of them had actual and thorough understanding of the objectives of the trial. They considered the clinical study aimed at providing good treatment and individual cure from the illness as quoted below:

‘I understand that I will get treatment and I will recover, by the grace of Allah. They told me many things but I didn’t understand everything’.

### Voluntariness

During the informed consent process, the study nurses who were responsible for obtaining consent read the patient information form (PIF) to all participants and also provided them with a copy of the PIF (see Additional file 2). All participants made a voluntary decision to participate in the TRAC trial. However, many admitted that they could not remember or understand everything that was explained to them because of their malaria symptoms during the informed consent process. In addition, most subjects (N = 15) were not aware of their right to quit the research anytime if they wished to do so. They rather wanted to continue after being enrolled as they had committed to this at the beginning. They also felt obliged to adhere to the study until the end so that they would get complete recovery from their malaria. One participant stated:

‘I don’t feel like quitting the study. Why should I? If I wanted to leave the study, I would say that. They told me at the beginning that I could only take part if I wanted to stay for 7 days. I agreed. I will be staying here till the end’.

There were mixed responses (8/16 subjects thoughts of not having treatment) related to perceptions of the availability of treatment in hospital if anyone declined to participate in the trial or withdrew consent after being enrolled. 

‘It depends on the doctors. If I said I was afraid to participate, they might not treat me’.

‘I would get treatment from hospital. Everyone is getting treatment here’.

### Factors influencing participation

The major factors influencing subjects’ decisions to participate in the research were availability of free medical treatment, access to good quality treatment and nursing care and financial support to compensate loss of work during the inpatient hospital admission. Some responses are listed below:

‘You have given me good care, I will tell others about this. I will tell them about the availability of good treatment here and the benefits I have received’.

‘If I wanted to get treatment from outside, I had to buy medicine. I wouldn’t get good medicine. There could be chance of getting malaria again. I think I have got good medicine here for many times’.

‘If I get treatment from outside, I won’t get well. Here in the hospital, treatment is free. I had to spend a lot of money to buy medicine for having treatment outside. I am poor, that’s why I have come here.’

The trial participants expressed high satisfaction with both the treatment received and the nursing care. They pointed out the availability of good quality treatment, free medication, and cure from the illness as major advantages of taking part in this trial. Most patients didn’t mention any inconveniences of being a research participant. Many thought blood samples were collected several times to investigate their disease progress, appreciated inpatient hospital admission and good nursing care. To the respondents, the most important aspect of their research participation was ‘to get well from malaria’.

*‘They can take whatever they want if I get well. If they take my flesh, I have no problem….* The interviewer asked whether there was any problem with long hospital stay. The subject said *“no problem at all”.’*

### Concept of being informed

Almost all subjects thought blood samples were taken for further investigations at home and abroad for the betterment of the individual patients. They were not interested in further details of the type of research (such as pharmacokinetic or genetic studies) the samples might be used for. No one found it important for the study team to ask for permission again prior to sending samples abroad for future research; instead they stated that this could be solely the researcher’s decision. Similarly, the respondents did not have a clear idea about further use of information collected through this clinical trial. The subjects were grateful to the study team due to having good treatment and nursing care, and none felt like asking any questions about research procedures.

‘I do not need to understand well. I am cured from my disease and I am happy for this’.

## Discussion

Informed consent is pivotal in protecting participants’ rights in human research. However, it is a challenge to ensure subjects are adequately informed and actually understand research goals and methods and able to make a voluntary decision in taking part or declining participation. Clinical trial guidelines do not explicitly define actual understanding of informed consent. Thus documentation of comprehension is not required. This exploratory study highlighted a ‘therapeutic misconception’ in which the participating patients viewed research activities as treatments aimed at managing their medical condition. From the patients’ perspectives, getting well from their illness was the major concern and participating in the trial was considered as the best treatment option. These particular subjects had no previous exposure to research and very little experience of institutional health care. The issue of research-treatment distinction has long been debated in bioethics [11-13]. Some argue that clinical research (whose goal is to generate generalizable knowledge) and medical care (focused on personalized care) should have clear-cut boundaries and are concerned about the dangers of mixing clinical research with treatment [14,15]. The potential for availability of free treatment and greater access to good quality treatment and care through research activities which are not available otherwise in a resource constrained setting raise an ethical dilemma, particularly if an investigational new drug is being evaluated, which was not the case here where the treatment was in line with the national standard of care.

Poor understanding of trial specific procedures was also reported despite participants’ satisfaction with treatment and nursing care. Similar findings have been observed in other developing and non-developing country settings [6,16]. In India, a study assessing comprehension of informed consent concluded that the comprehension could be reasonably good providing the consent form was explained in simple language to the participants [17]. A recent article involving oncology patients at the University of Wisconsin, Madison, found that one-fifth of subjects had considerably less understanding of study aims, methods and risks [18]. It has increasingly been acknowledged by the scientific community that the lengthy, complicated informed consent process needs to be adapted and a shorter, more understandable consent form will better enable participants to understand the implications of their research participation, benefits, risks and obligations; to have clear, complete information while making a decision. The informed consent process is in danger of becoming a box-ticking exercise focused more on offering legal protection to a trial’s sponsor and illustrating GCP compliance rather than its original intention of protecting participants and ensuring comprehension of study information [19].

Limitations of this research study are the small sample size and the lack of characterization of those patients who refused to participate. However some clear themes did emerge even with this number of subjects. This study was not designed to measure consent comprehension directly but rather aimed at gathering evidence on subjects’ understanding and perceptions of research. Consent comprehension is a complex issue. Bloom’s Taxonomy of Educational Objectives emphasized six levels of cognitive skills related to informed consent: knowledge, comprehension, application, analysis, synthesis, and evaluation [20]. Understanding is a complex process containing cognitive, logical and emotional components. Understanding requires memory but memory does not require understanding. Memory capacity and overload are a concern in a trial like this. Consent forms usually include at least eight ‘required elements’ and six ‘when appropriate’ elements. Memory research shows that people can hold about 3–5 items in their working memory [21]. It is unrealistic to expect subjects to remember much from a prolonged consent discussion with an information sheet stretching to several pages, especially when they are unwell. Another important issue is readability, even more complex in a developing country where many of the potential participants are illiterate. The regulatory authorities and Institutional review boards (IRBs) recommend that consent forms should be written at a sixth-eighth grade reading level. Readability alone does not ensure understanding. Comprehension will be lower if consent forms contain many words that are familiar to researchers but unfamiliar to subjects. Subjects’ reading skills, existing knowledge of clinical trials, their interest or disinterest in reading the complete form are important considerations.

There are inconsistent research findings in the literature on the effectiveness of strategies designed to improve understanding and consent comprehension [22]. Several intervention strategies and tools, such as multimedia technology (video and computer interventions), enhanced or modified consent forms (shortened form, improved readability, revised content and improved formatting), and an additional person-to-person interaction after the initial formal standard consent process have been assessed and showed a range of effectiveness in specific research contexts [23]. It was concluded that longer contact between a researcher or a nurse or a neutral counselor and a prospective subject was the only method which appeared to produce consistent comprehension improvements. Malaria patients usually have fever and are unwell when they are recruited to a clinical study and it may be that a second discussion 24–48 hours after enrolment to explain the aims of the study again and the meaning of their consent would result in improved comprehension. It is also noteworthy that none of these participants had any prior experience of research. The rates of patient refusal to participate to the clinical trial itself were several-fold higher at other sites where patients were research-experienced (E Ashley personal communication 2014).

## Conclusions

This small-scale study described the baseline understanding of subjects enrolled in a malaria research trial in Bangladesh. The findings indicate a need to improve the informed consent process in regards to information provision and study comprehension. A second discussion when patients have recovered from their presenting symptoms of acute malaria might lead to improved comprehension and more informed consent. In many resource constrained settings, research participation offers improved access to quality health care services and free treatment which are either not available or perceived to be unavailable in routine medical care, meaning potential participants are more vulnerable and less likely to refuse to take part. Another way to improve the safeguarding of patients’ interests may be to engage the community prior to the research [24-26]. This study was an initial attempt to have a better understanding of research participants’ perceptions and understanding of research and to determine factors influencing their decision making process. The findings suggest that use of a standard consent form following the current ICH-GCP guidelines does not result in achieving fully informed consent and the process should be revised, simplified and adapted to individual trial settings.

## Competing interests

The authors declare that they have no competing interests.

## Authors’ contributions

The study design and questionnaire were developed by AF, NJW, EA, PYC, AG, and DD. Interviews were carried out by FA and DD. Recorded interviews were then translated from Bangla to English by DD. The transcripts were read by DD, PYC and EA. Emerging themes were discussed and agreed by all team members. DD wrote the first draft of the manuscript. EA, PYC, and AF reviewed and edited the manuscript. All authors read and approved the final manuscript.

## Supplementary Material

Additional file 1 Interview guide. Click here for file

Additional file 2 TRAC Patient Information Form. Click here for file
