# Supplementary material for: Participants’ perceptions and understanding of a malaria clinical trial in Bangladesh
Source: Malar J. 2014 Jun 4;13:217. doi: 10.1186/1475-2875-13-217 (PMC4055798; doi:10.1186/1475-2875-13-217)
Supplement: Additional file 1 — Interview guide. [file 1475-2875-13-217-S1.pdf]

Interview Guide

1. Is this the first time you have been involved in malaria research?
2. How many times had you been involved in malaria research in the past?
3. Tell us about your current illness starting from the onset of illness to arrival at hospital and your experience of taking part in this research.
4. You had to give repeated blood samples. What could be the reasons?
5. What may be the reason (s) for your admission to hospital?
6. Based on your understanding, how many days more antimalarial medicine will you receive?
7. What is your impression about the objectives of conducting the TRAC trial?
8. Overall, do you think that doing the TRAC study in your country is… (Very beneficial/Moderately beneficial/Neither beneficial nor harmful/Moderately harmful/Very harmful/Don't know)
9. Did you know that you were free to decline to participate in the TRAC trial?
10. Are you allowed to quit the TRAC trial if you want to?
11. When are you allowed to quit the study? Are you allowed to quit…(Anytime you want/Only after you receive the first dose of the antimalarial treatment/Only when the study ends/Or only when the doctor says you can quit/Don't know)
12. If you quit the study, will you still be treated for malaria in the hospital?
13. If you decided not to take part in the research, how could you manage to get treatment for malaria?
14. What were the advantages of taking part in the research?
15. What were your motivations for participation in this research?
16. What were the inconveniences of being a research participant?
17. What will happen to the blood samples taken from you?

18. How important is it for you to know what kind of research your blood sample will be used for? (Very important/Moderately important/Slightly important, or/Not important at all/Don’t know)
19. Before using your blood sample for a specific research study, how important would it be to you to have the TRAC study team get your consent first? (Very important/Moderately important/Slightly important, or/Not important at all/Don’t know)
20. What will happen to the information collected from you?
21. How would you be benefited individually from the outcome of this research study?
22. Was the information disclosed to you during consent process informative and understandable?
23. Did you receive a form? Tell me about the information sheet. How long was it?
24. Did you read (if a patient unable to read, listening to somebody) the information sheet that you received from the study team?
25. Did you feel comfortable to ask questions or to ask for information to be repeated?
26. Do you have any opinions or suggestions on improving the informed consent process?
