# Supplementary material for: Participants’ perceptions and understanding of a malaria clinical trial in Bangladesh
Source: Malar J. 2014 Jun 4;13:217. doi: 10.1186/1475-2875-13-217 (PMC4055798; doi:10.1186/1475-2875-13-217)
Supplement: Additional file 2 — TRAC Patient Information Form. [file 1475-2875-13-217-S2.pdf]

PARTICIPANT INFORMATION SHEET

Note: If you are a parent or guardian of a child below 18 years old, please read "you" as "your child".

You have been diagnosed with falciparum malaria. Malaria is caused by parasites which infect the blood and is transmitted by mosquitoes. We are conducting a study to determine how well an antimalarial drug called artesunate is working in treating malaria. We would like to ask you to participate in this study.

In this leaflet, we will give you information about the study to help you decide whether or not you want to take part. If you have any questions or concerns, you will have a chance to discuss them with the study doctor.

What is the study about?

• We are studying a drug called ARTESUNATE in about 1800 patients who have been diagnosed with falciparum malaria around the world.

• ARTESUNATE is an artemisinin type drug. These drugs are recommended and used all around the world to treat people with malaria. ARTESUNATE usually works very quickly and is very safe and effective at curing the disease.

• In 2009, a study found that in some parts of Cambodia ARTESUNATE was not working as quickly as before - it was taking a longer time for malaria parasites to disappear from the blood after treatment. We call this problem artemisinin “resistance”.

• We are now conducting a similar study here and several other countries in Asia, as well as 2 countries in Africa. We want to measure how quickly the parasites disappear from your blood after you have been treated with ARTESUNATE.

• This is important to know, because new treatments might be necessary and we need to know how far this problem of artemisinin resistance has spread.

Do I have to take part?

• No. You should only take part if you decide you want to. We want to give you enough information to help you make an informed decision. Once you have this information, it will be your decision to take part or not.

We will assess you to see if you can take part in this study. You will be asked a number of questions about your health, both now and in the past, been examined and have blood taken. If you are female, please tell us if you are pregnant or think you may be pregnant.

• If you do not agree to take part in the study or we find you are unsuitable, you will be given the standard treatment for your malaria. If you agree to the study but later change your mind, you can withdraw without giving any reason, without affecting your treatment.

• You may ask questions at any time. You may also want to talk to your family and friends about your decision.

What will happen if I take part in the study?

• You will need to stay in hospital for at least 3 nights while you are treated and observed.

• It is important for you stay in hospital to be sure the malaria had gone from your body. For some patients, this may mean staying in hospital for more than 3 nights.

• A doctor will examine you on the day you are admitted and regularly during your stay in hospital.

• We will need to take a sample of your blood when you arrive, and also a number of times during your stay.

• You will then be given one of the two doses of ARTESUNATE, either 2mg/kg or 4 mg/kg for 3 days. The dose of ARTESUNATE you receive will be determined at random by lottery, so that we will not know in advance which dose you will receive. This will be followed by a course of anti-malarial treatment that is routinely used in your hospital or country.
(Note: In Western Cambodia, only 4 mg/kg will be given.)

What does giving blood involve?

• We will put a thin plastic cannula into a vein in the back of your hand or your forearm. We will use this to collect small amounts of blood. A cannula will be in place for about 24 hours so you will not need to have a pin-prick every time you give blood.

• When you first arrive, the extra blood needed for the study will be up to 3 mL (up to 1 mL for children). One teaspoon is 5 mL of blood.

• After admission sample, we will collect small drops of blood to look for malaria in your blood and measure your red blood count at 4, 6, 8, 12 hours then 6 hourly until you are discharged from the hospital. We will also take blood for special tests on the malaria itself and measure the artesunate in your blood.

What will the blood sample be used for?
• The blood sample will be used to assess how quickly the malaria is cleared from your blood. In addition to this, the blood sample will be used to see if your blood test if you have any types of blood that resistance of the parasite to antimalarial drugs, to measure drug concentrations, and to do genetic studies on the parasites to try and explain why they can resist the drug. For this reason, the samples will be transferred to the laboratories of Mahidol-Oxford Tropical Medicine Research Unit in Thailand and sent on to other specialist laboratories in other parts of the world e.g. UK, USA.

If you consent some of your leftover blood samples will be stored and may be used for further studies. Any new tests not covered in the present protocol will not be carried out unless a separate approval is obtained from the relevant ethics committees.

How much blood will be given and is it harmful to give this amount of blood?

For adults, the maximum blood volume will be 60 mL (14 days of follow up) and 62 mL (42 days of follow up, less than 4 tablespoon). For children, the corresponding volumes are 46 and 48 mL (4 tablespoon). Allowing for the possibility that we may need to repeat blood tests, we will add 2 mL to these maximum blood volumes.

• It is not harmful to your body as your body can easily cope with giving this much blood. It will not affect your health, and most people do not feel any different after giving this amount of blood.

• Inserting the cannula into your vein can be uncomfortable at first (like a pin-prick or a sharp scratch). Once it is in place, it is not usually painful.

• Sometimes when a cannula is inserted, it can cause a small amount of bleeding into the skin, causing some bruising and mild discomfort. To keep the risk of this as small as possible, well-trained and experienced doctors and nurses will collect the blood.

• Sometimes the tissues around the cannula can become inflamed, or the cannula can become displaced and stop working. In this case, we may need to take the cannula out and put one into another vein as a replacement.

What will happen after I leave hospital?

• After you return home, you will need to visit the hospital clinic for your follow up treatment and assessment on Days 4, 5, 7 and 14. If it is required by your hospital or your doctor, you need to come to the clinic weekly until for up to Day 42. At each visit, we will check that you are still in good health, and take a small amount of blood to make sure that the malaria parasites have not come back.

• We will pay for a reasonable amount of travel costs, and for your lost time in making these visits.

• These visits are important for the study, as they allow us to make sure the disease is really cured. They will also give us a chance to make sure you are still feeling well.

-If you agree, we might also ask to visit you at home so that we can observe the environment and understand the risks for malaria.

What will happen to the information you collect?

• The information we collect from speaking to you and from analyzing your blood samples will be kept confidential by the study team. We will not share your personal information with anyone outside the study. No one other than the study team and authorised personnel from the

study sponsor and regulatory authorities are allowed direct access to your personally identified medical records.

• When the study is completed, we will combine your test results with those of the other participants, and the overall results will be analyzed.

• We would like to share these overall results, including with a group called the World Wide Anti Malarial Resistance Network (WWARN) and the World Health Organisation (WHO) who are monitoring how well antimalarial drugs are working worldwide. The National Malaria Control Programme in your country will be responsible for how these data are used. We will also publish the results in the medical literature. In both these cases we will make sure that you cannot be personally identified.

What are the advantages of taking part?

• If you decide to take part, we will treat your disease, but we will be happy to do this even if you decide not to take part. In either case, the drugs we use to treat your malaria (and any other drugs you need) will be given to you for free.

We will monitor you closely to make sure you have cleared the malaria from your blood

• Although you may not get a direct benefit from taking part, the results of the study will improve our understanding of malaria in your area, and whether artesunate is still working well or not.

What are the disadvantages of taking part?

• You will need to stay in the clinic for at least 3 nights and then come back once daily for 3 days to receive the rest of the treatment. You will also need to come to the hospital once a week for at least 2 weeks after you return home.

• You will need to give blood more frequently than would usually be required to treat your disease. Although this may be uncomfortable, we do not expect it to be harmful to your health. During the first 3 days when frequent blood taking will be required, you will not be able to leave the hospital compound. Therefore, you may need to make arrangements with your relatives/friends to help you.

ARTESUNATE is very well tolerated, side effect that are common are mild diarrhoea and abdominal pain. Serious hypersensitivity reaction can occur rarely. Otherwise, at the doses used for the treatment of acute malaria, there are no known toxicities. The other malaria medicines used in this study have been studied thoroughly and used routinely to treat malarial and their toxicities are well described. In general, they are all well tolerated.

• If you do have any side effects, or any unexpected problems during your stay with us, we will treat these problems fully and with no charge to you or your family. In the unlikely event that you are harmed as a direct result of participation in the study, compensation will be available from the University of Oxford's insurance scheme.

• We appreciate that taking part in this study may be inconvenient for you and your relatives. We will also give you a small amount of money to compensate you for your lost time and to cover the cost of food.

What if I decide not to take part?

• You do not have to take part in this study. You can also stop at any time after the study has started.

• If you decide not to take part, or if you withdraw after the study begins, we will still treat your illness for free. You can withdraw from the study for any reason, and this will not affect your further treatment.

Who is conducting this study and who has approved it?

The study is conducted by a group of doctors and scientists in the Tracking Resistance to Artemisinin Collaboration (TRAC). It is sponsored by and insured via the University of Oxford and funded by the UK Department for International Development. The study has been approved by the Oxford Tropical Research Ethics Committee and the ethics committees in your hospital/country.

What if I have questions?

• If you have any questions or concerns after reading this leaflet, you will have a chance to discuss them fully with a member of our team before you decide whether or not to take part. We will also be available throughout the study to answer any questions or address any concerns that you may have later on.

• While you are in hospital, you can contact your attending doctors and nurses 24 hours a day with any questions or concerns.

• If you have any questions while you are at home, you can contact xxxxxx by telephone at xxxxxxxxx.
